# Morpho-physiological responses of tall wheatgrass populations to different levels of water stress

**DOI:** 10.1371/journal.pone.0209281

**Published:** 2018-12-17

**Authors:** Celina I. Borrajo, Adela M. Sánchez-Moreiras, Manuel J. Reigosa

**Affiliations:** Department of Plant Biology and Soil Science, Faculty of Biology, University of Vigo, Vigo, Pontevedra, Spain; Swedish University of Agricultural Sciences, SWEDEN

## Abstract

Tall wheatgrass [*Elymus elongatus* subsp. *ponticus* (Podp.) Melderis] is a perennial forage grass cultivated in dry, saline or alkaline environments. The morpho-physiological characteristics of four populations of tall wheatgrass from different climatic-edaphic origins were evaluated under three conditions of water stress (100%-50%-30% of field capacity). The trial was analyzed with three replicates and two-factor ANOVA in pots within the greenhouse during 35 days. Only dry matter and tiller number showed interaction between populations and water conditions. The most relevant changes in morpho-physiological parameters under strong water stress were reduced dry matter production (48–32% differing among populations), smaller leaf and tiller size (46% and 28%), together with higher water use efficiency (74%), and increased proline and protein contents (144% and 71%), smaller tiller number (30–11% differing among populations) and a slight decrease in leaf water content (3%). The populations differed in growth strategies and morpho-physiological mechanisms to survive water stress, which could be related to their habitat background. The study shows the stability in dry matter production under all levels of water stress, which can be related to the higher tiller number. Due to this plasticity, tall wheatgrass should be studied as a species with great potential to adapt to drought stress.

## Introduction

Tall wheatgrass [*Elymus elongatus* (Host) Greuter subsp. *ponticus* (Podp.) Melderis syn. *Thinopyrum ponticum* (Podp.) Barkworth & D.R. Dewey (2n = 10x = 70)], is a C3 *Poaceae* species, original from dry or saline habitats of Southeastern Europe [1]. Tall wheatgrass is a perennial grass of high phenotypic plasticity used as fodder for livestock feeding in environments with climate-edaphic limitations of Australia [2], Argentina [3–4], Canada and USA [5]. Also, tall wheatgrass has become important due to its genetic relationship with wheat and its potential as gene source for resistance to diseases and tolerance to abiotic stress [6–8].

Tall wheatgrass was introduced in Argentina during the 1950s. It is cultivated in more than 500,000 ha of the Depressed Pampa of Buenos Aires, Argentina, in temperate-humid climate under salinity, alkalinity, winter flooding, and summer drought conditions [3–4]. Currently, tall wheatgrass is naturalized in several environments in Argentina [4, 9], and displays agronomic variability among populations from different environments of the Pampa Region [10]. Due to this plasticity, tall wheatgrass should be studied as a species with great potential to adapt to climate change and may have great potential for increasing the forage production in saline and arid areas [3–4, 11].

Climate change will cause episodes of water deficit with greater frequency and intensity, reducing the productivity of agroecosystems. Water deficit in plants causes different responses depending on the intensity and duration of the stress, as well as variations according to species, genotype and phenological stages [12–13]. The response of the plant results in changes of growth, development, morphology, physiology, cell adaptations and osmotic adjustment mechanisms, such as the synthesis of solutes, as proline, or specific proteins, which are essential to overcome stress [14–16]. Water stress usually results in reduced protein content and increased proline in plants [17]. Some authors mention that proline accumulation could be a symptom of damage to the plant [17], but other authors link it to osmoregulation and osmoprotection mechanisms [12]. More recent reports show proline as a multifunctional amino acid essential for adapting to, recovering from and indicating environmental stress [16].

Grass breeding improvement programs seek to increase the aerial biomass production and drought tolerance. However, the main mechanism to moderate water use under drought stress is the reduction in the size of the plant, leaf area and tillering, swhich limit yield potential [15]. Germplasm evaluation of forage species for improvement purposes has been traditionally based on phenotypical characteristics, and the study of molecular, genomic or metabolomic resources has been limited [18–19]. More research on forage cultivars is needed to identify metabolic traits and pathways that confer drought tolerance, and this research should be integrated with plant physiology and genetics [14, 19]. These studies are not easily translated to perennial forage species as most of these metabolic analyses have been performed with cereals or annual species [19].

The evolution and genome composition of tall wheatgrass have been studied through comparisons of species relationships and hybridizations with wheat [7–8]. Nevertheless, the existence of intra-specific variability in water stress tolerance has not been widely explored for tall wheatgrass. Although, survival, morphology, production and quality parameters of tall wheatgrass genotype in saline, alkaline or neutral environments have been described [5–6, 10–11, 20–22], there are few research studies about the physiological mechanisms responsible for this behaviour [7, 23–25]. It is essential to evaluate the germplasm including the production and physiological perspectives, so that the characteristics of interest can be selected with greater precision. The knowledge of these responses in *Elymus elongatus* subsp. *ponticus* would provide new tools to discover the most adapted germplasm to water stress imposed by climate change. Therefore, the main objective of this work was to evaluate the impact of water stress on the morpho-physiological characteristics of *Elymus elongatus* subsp. *ponticus*, analyzing populations from different climatic-edaphic origins during 35 days.

## Materials and methods

The **e**xperiment was conducted in pots at the greenhouse located at the Campus Lagoas-Marcosende of the University of Vigo, Spain (42°10’0.38”N, 8°41’3.37”W), with natural light (15/9 h light/darkness) and an average air temperature of 22.7°C, with maximum temperatures of 30.0°C and minimum temperatures of 15.5°C (day/night). Water stress was established at different water levels, based upon the field water capacity for each pot. Field water capacity was estimated according to the definition of Soil Science Glossary Terms Committee [26] as the content of water, on a mass or volume basis, remaining in the soil 2 or 3 days after having been wetted with water and after free drainage is negligible. Field capacity in pot was considered as the content of water of the soil humid portion, after the excess of water has been drained (waterlogging) and the loss speed has been reduced to a significant degree (100% water at field capacity). Three water levels (WL) were established in 100%, 50% and 30% of field capacity, simulating three levels of water stress: no stress, moderate, and severe stress (WL: 100_WL_, 50_WL_, 30_WL_, respectively). The water levels were maintained by weighting the pots every 2.5 days, and adding the amount of water lost by evapotranspiration (adapted from [27]).

The naturalized populations of tall wheatgrass [*Elymus elongatus* (Host) Greuter subsp. *ponticus* (Podp.) Melderis syn. *Thinopyrum ponticum* (Podp.) Barkworth & D.R. Dewey (2n = 10x = 70)] were provided by the Active Germplasm Bank of Balcarce Agricultural Experimental Station of the National Institute of Agricultural Technology, Argentina (BAL). In this work, four populations (labeled P3, P4, P5, P9 [Table 1]) were selected from a large seed collection, considering the habitat backgrounds and the availability of seeds from BAL. Populations were selected by contrasting clima-edaphic environments of the Argentine Pampa, from semi-arid to temperate oceanic climate, and alkaline and neutral soils. Seeds were germinated in chambers (30°/20°C and 8/16 h light/darkness), and the seedlings were grown in greenhouse in small pots with peat as substratum (Compo Sana Universal R peat), for 24-days prior to being transplanted to test pots.

**Table 1:** Collection data of tall wheatgrass populations from Active Germplasm Bank of Balcarce Agricultural Experimental Station of the INTA, Argentina (BAL).

Population	P3	P4	P5	P9
**Collection BAL**^**a**^	Nu+Alo338	CIB118	CIB117	CIB114
**Nearest town,**	Necochea,	Lamarque,	Lamarque,	Bahía Blanca,
**province.**	Buenos Aires.	Río Negro	Río Negro	Buenos Aires.
**Latitude**	38°30’S	39°24'S	39°24'S	38°44'S
**Longitude**	58°45’W	65°36'W	65°36'W	62°33'W
**Climate**	Temperate oceanic	Semiarid	Semiarid	Temperate transitional
**Soil** ^**b**^,	*Argiudolls*	*Torrifluvents*	*Torrifluvents*	*Haplustolls*
**pH.**	Neutral, pH ≈ 7.0	Alkaline, pH: 9.0	Neutral, pH: 7.5	Alkaline, pH: 9.5
**Precipitation**	900 mm year^-1^	300 mm year^-1^	300 mm year^-1^	500 mm year^-1^
**Environment**	Roadside grassland.	Grassland, with *Distichlis spicata*	Roadside of irrigated fields, 450 mm year^-1^	Grassland, with *Distichlis spicata*.

^a^ Collector's code (Nu, Alo, CIB) and entry number.

^b^ Great Group, Soil Taxonomy.

The experiment was laid out as a randomized complete block design with three replicates and two factors. One factor was the four populations (Popu) and the other was the three water levels (WL). The experimental unit was a pot. Each pot (1 L) was filled up to three-quarters of its capacity with peat as substratum (Compo Sana Universal R peat), and transplanting three seedlings of one tiller per pot. The experiment was started with plants in the seedling stage and finished with the plants in vegetative state. The duration of the experiment was set at 35 days because the largest area of tall wheatgrass pastures, which is found in the Depressed Pampa [3–4], shows temporary droughts of less than one month.

### Non-destructive measurements

Two non-destructive variables were periodically measured. The number of tillers per plant (3 plant per pot) was recorded every week, totaling 4 records (time = 4). The tiller number was expressed in tillers pot^-1^.

Evapotranspiration (ET) was calculated in each pot and was estimated using the equation:

ET=[(Irrigatedpotweight)−(Postirrigatedpotweight)]

ET was calculated as the difference between the weight of the newly irrigated pot and the weight of the pot 2.5 days later. ET was measured in g _H2O_ pot^-1^ and was expressed in mL_H2O_ pot^-1^. ET was recorded in 13 different times.

Accumulated evapotranspiration was then calculated as

$$ETAccu=\sum_{ET=1}^{13}ET$$

### Postharvest measurements

Once the experiment was finished, the aboveground biomass was harvested and the following parameters were measured. The production of fresh and dry matter (FM and DM, g pot^-1^), where FM is the fresh weight of the forage and DM is the weight estimated after drying the forage at 50°C to constant weight. Leaf water content was estimated using the equation:

$$LWC\;(\%)=\frac{\left(FM-DM\right)}{FM}*100$$

Water use efficiency was expressed in mg mL^-1^_H2O_ and was calculated using the equation:

$$WUE=\frac{DM}{ETAccu}$$

Tiller weight was expressed in g tiller^-1^ and estimated using the equation:

$$Tiller\;weight=\frac{DM}{Density}$$

where Density is the tiller number per pot in the 4th week.

Leaf blade length (cm), width (cm) and area (cm^2^) were recorded from the last developed leaf of 3 tillers per pot (Llength, Lwidth, Larea, respectively); using the ImageJ program [28]. The specific leaf area, SLA (cm^2^ g^-1^) was estimated based on the ratio between area and dry mass of the leaves.

Two samples of leaves were also frozen at -80 ºC to analyze the content of soluble protein (100 mg FM) and free proline (250 mg FM). Protein content quantification was determined by the Bradford method and expressed in mg g^-1^_DM_ [29]. Free proline content was determined according to the Bates method and expressed in μmol g^-1^_DM_ [30]. Furthermore, the relative proline content (Pro Rel) was calculated as the difference in the concentration of free proline between the stress treatment and the control, using the equation:

$${ProRel}_{WLx}=\frac{\left({\left[Proline\right]}_{WLx}-{\left[Proline\right]}_{100WL}\right)}{{\left[Proline\right]}_{100WL}}*100$$

where [Proline] is the proline concentration, WLx is the stress treatment: moderate (50_WL_) or severe (30_WL_), and 100_WL_ is the control (unstressed plants).

### Statistical analyses

Dry matter, accumulated evapotranspiration, water use efficiency, tiller weight, length, width and area of the leaf, leaf water content, specific leaf area, free proline and soluble protein were analyzed with two-factor ANOVA. Tillers were analyzed with two-factor ANOVA and measures were repeated over time. Block was used as random effect. The comparisons of means were made with least significance difference (LSD) test (LSMEANS statement). In all cases a 5% probability level and Proc Mixed were used [31]. ET_Accu, proline, protein and tiller number did not present variance homogeneity and normally distributed data, so they were logarithmically transformed to be analyzed. The Pearson correlation coefficient was calculated in some pairs of variables, using the corr procedure of SAS.

## Results

Water level was the variable that affected the physiological parameters measured in this study most, with significant differences in all of them (p < 0.05). The four populations responded differently to water stress showing different values for dry matter, water use efficiency, tiller weight, leaf width and area, leaf water content, proline and protein contents and tillers (p < 0.05), and similar values for accumulated evapotranspiration, leaf length and specific leaf area (p > 0.05). Only dry matter and Tillers showed significant interaction between water levels and populations (p < 0.05, S1 Table and S2 Table). At the end of the experiment plants were in vegetative state developing leaves and tillers for all water levels and populations.

### Dry matter production

**Fig 1:**
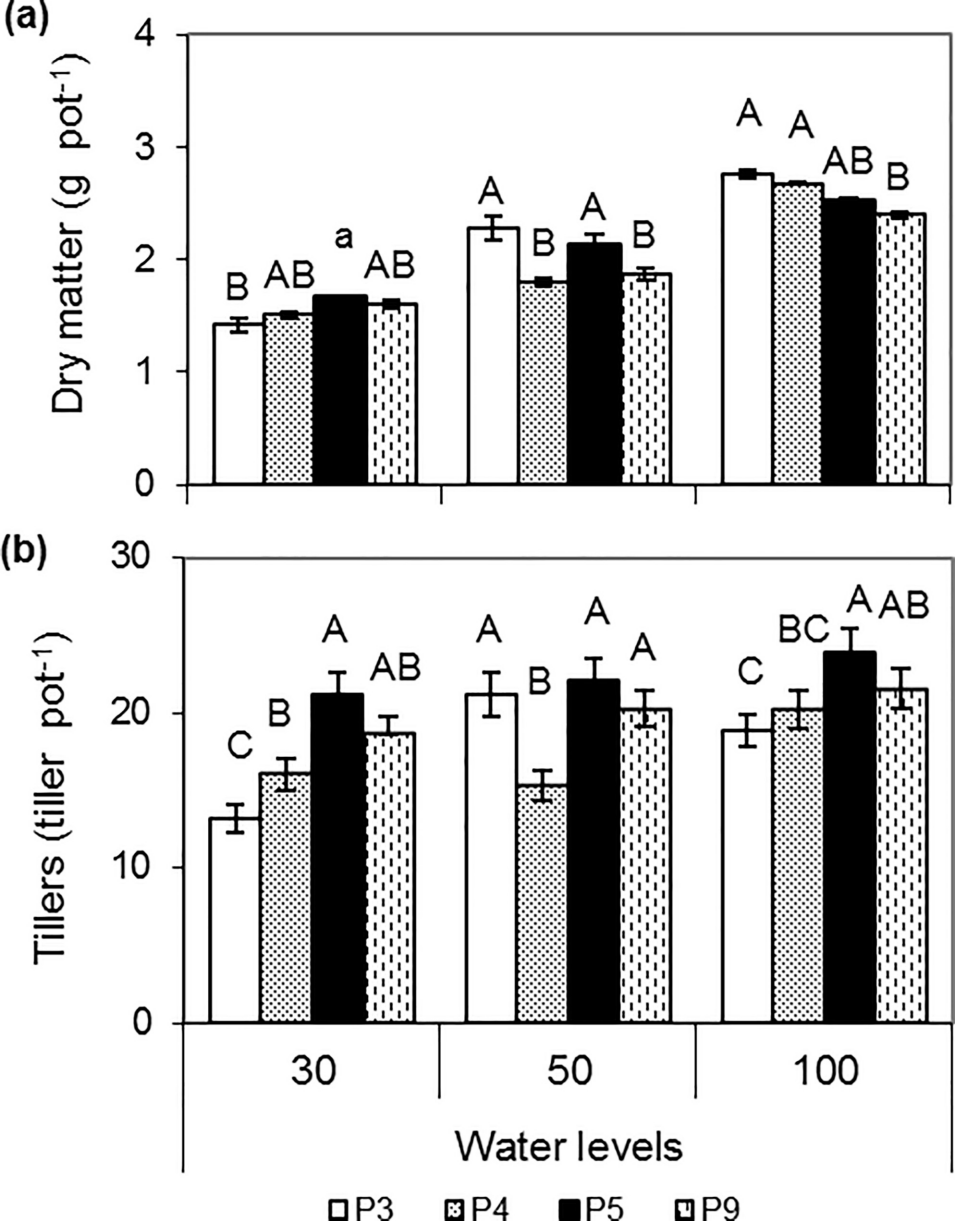
Variation of dry matter production (a) and tiller number (b) according to interaction between water levels and populations (P3, P4, P5, P9). Bars with different letters show significant differences (P < 0.05) among populations for each water level (n = 3 in DM and n = 12 in tillers). The vertical line above the bar indicates the mean standard deviation.

In general, dry matter values decreased due to the restriction of water levels, but populations responded differently to water levels (interaction WCxPopu p = 0.0003). The interaction was examined by comparing the means among populations at each water level (Fig 1A). P3 was the population with the highest values of dry matter in no stress and moderate stress levels (100_WL_ and 50_WL_), but with the lowest in values of severe stress (30_WL_). P5 was the population that grew most under severe stress, significantly differing from P3. However, no differences were found between P5 and P3 under moderate stress or optimal conditions (no stress). P4 and P9 were the populations with the lowest values of growth under moderate water stress, while showed intermediate values under severe stress. However, growth of these populations differed under optimal conditions as P4 showed low values and P9 high values of growth. Dry matter production under severe stress was reduced depending on the populations as follows: 48% in P3, 44% in P4, 34% in P5 and 32% in P9, compared to DM under no stress.

### Number and weight of tillers, and leaf size

Tiller number of each population responded differently to water levels (interaction WCxPopu p < 0.0001). As showed by the means comparison among populations at each water level (Fig 1B). P5 developed more tillers per plant at all water levels, while P3 showed the lowest number, except for plants grown under moderate stress, where P3 did not differed significantly from P5. Tiller number under severe stress decreased depending on the population as follows: 30% in P3, 21% in P4, 14% in P9 and 11% in P5, compared to the tillers in no stress. In general, considering the significance in the interaction and the % reduction shown in the tiller number, P9 was more similar to P5, while P4 was closer to P3. The amount of tillers increased with the duration of the experiment, with significant differences among weeks (S1 Fig).

**Fig 2:**
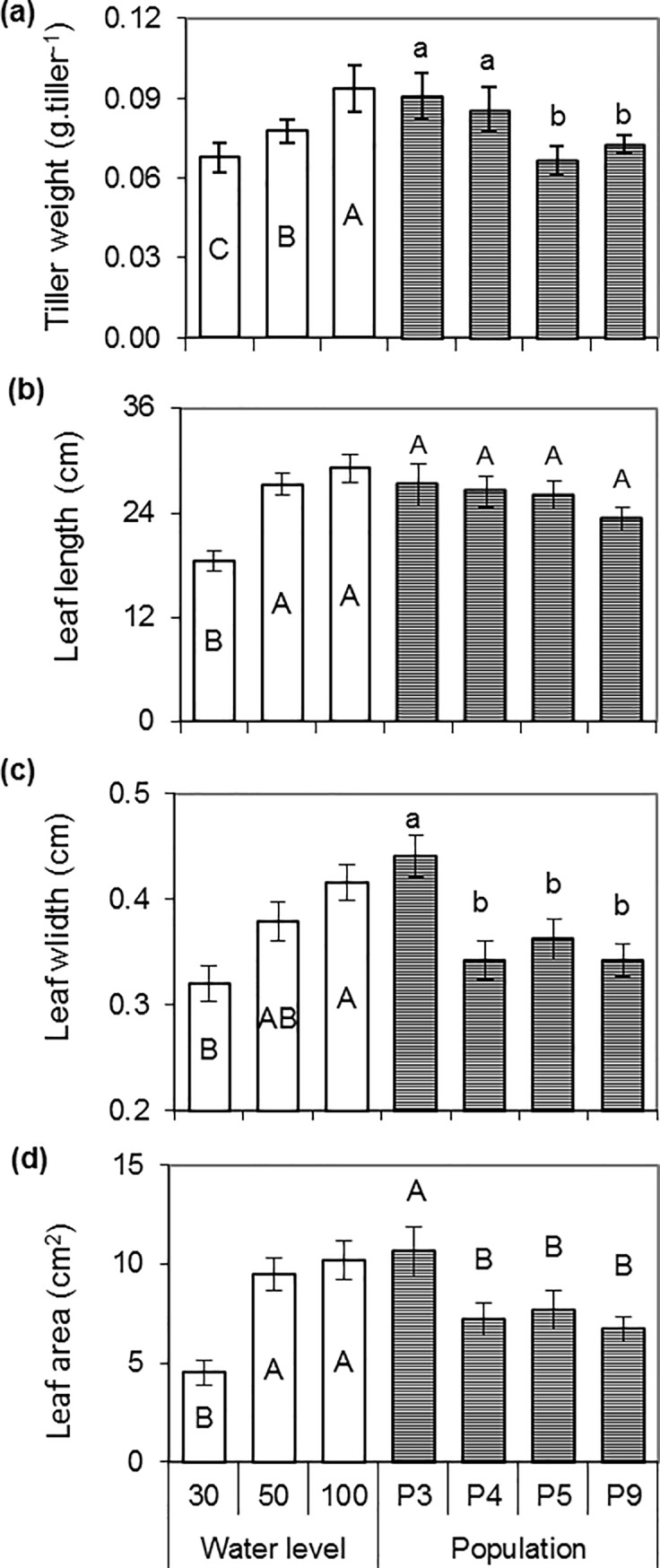
**Variation of tiller weight (a), leaf length (b), leaf width (c), leaf area (d) according to water levels and populations.** Bars with different letters show significant differences among means (P < 0.05), populations (n = 9) or water level (n = 12). The vertical line above the bar indicates the mean standard deviation.

Tiller weight was significantly lower at higher water stress levels, decreasing 28% from 100_WL_ to 30_WL_. P3 and P4 populations had tillers with greater weight than P9 and P5 (Fig 2A). Leaf length, width and area were positively related to water level. The control treatment (100_WL_) showed the highest values which did not differ significantly from moderate stress (50_WL_). However, under severe stress (30_WL_) leaf length, width and area decreased 40%, 23% and 46% respectively, compared to control (Fig 2B, 2C and 2D, respectively). P3 showed significantly higher leaf width and area and leaf and tiller size, standing out among the populations, while P5 showed the highest tiller production at all water levels.

**Fig 3:**
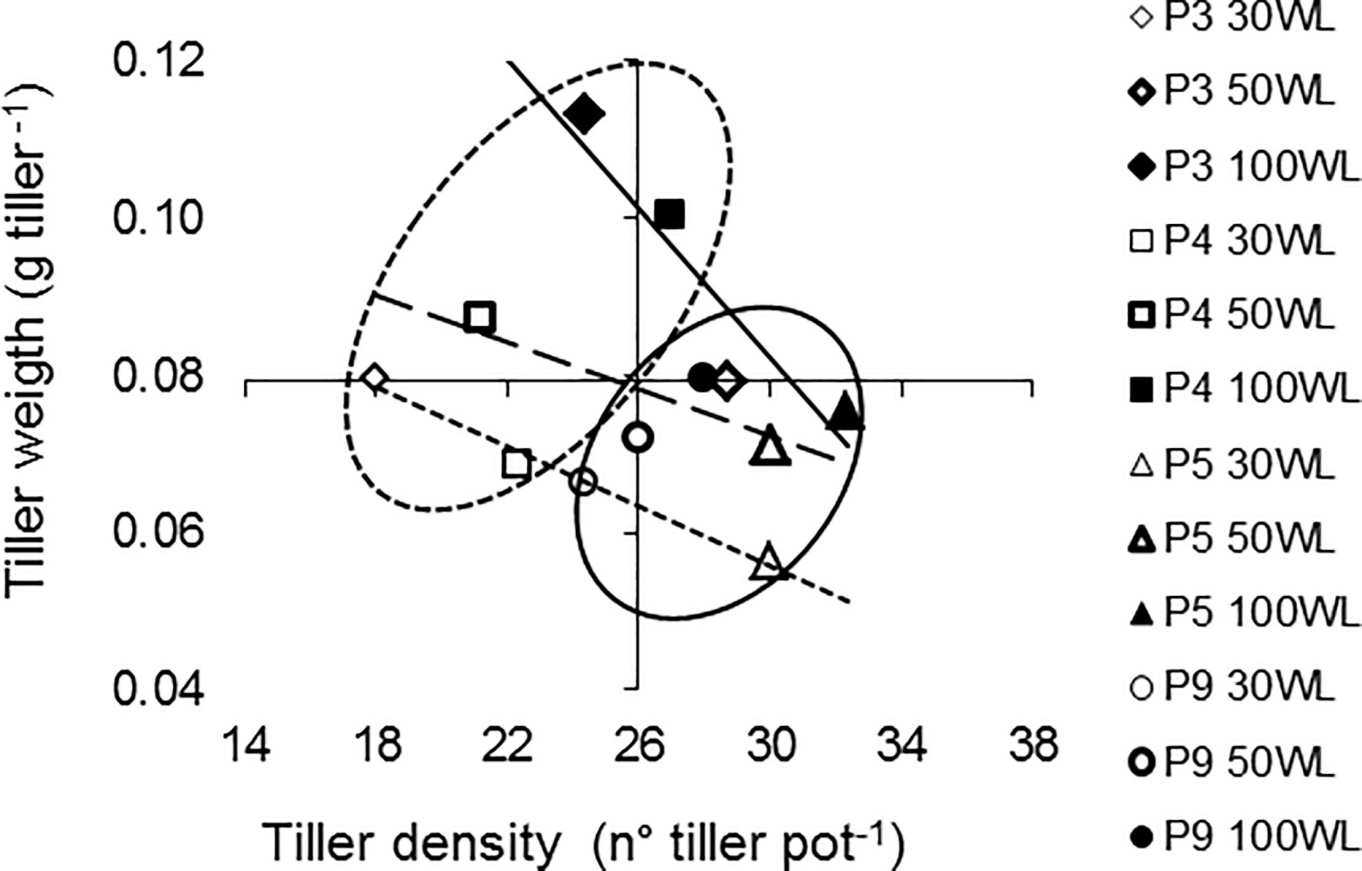
Relationship between density and weight of tillers (axes in average values) for the populations (P3, P4, P5, P9) under different water levels (WL: 100, 50, 30). Each symbol is the mean of n = 3. The circles enclose populations with similar canopy morphological pattern (cut line, P3 and P4 with bigger size and fewer tillers, and continuous line, P5 and P9 with smaller size and more tillers). The lines between density and weight of tillers show the response of populations watered with the same water level (100WL continuous line, 50WL cut line and 30WL dotted line).

Considering the germplasm assessment, two groups could be established according to canopy morphological patterns: P3 and P4 with larger weight and lower number of tillers, and P5 and P9 with smaller weight and higher number of tillers (Fig 3).

### Evapotranspiration and water use efficiency

**Fig 4:**
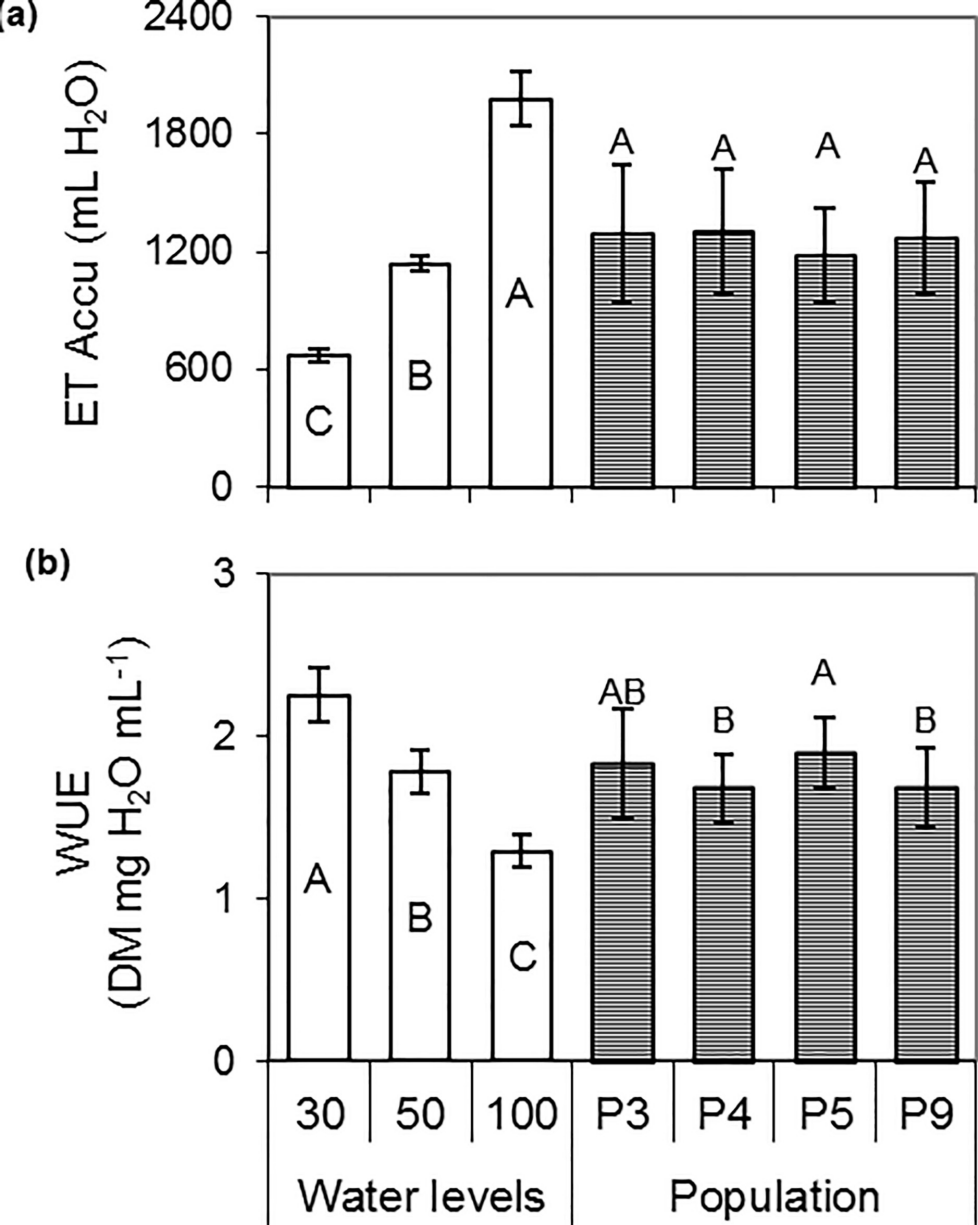
**Variation of accumulated evapotranspiration ET_Accu (a) and water use efficiency WUE (b) according to water levels and populations.** Bars with different letters show significant differences among means (P < 0.05) for populations (n = 9) or water level (n = 12). The vertical line above the bar indicates the mean standard deviation.

Accumulated evapotranspiration increased with water level (19, 33 and 57 mL day^-1^ in 30_WL_, 50_WL_ and 100_WL_, respectively), but no significant differences were found among populations (Fig 4A). Water use efficiency was negatively related to water availability, with maximum values in plants grown under severe stress (30_WL_). Among the populations, P5 showed the highest values but did not differ significantly from P3 (Fig 4B).

### Physiological parameters

**Fig 5:**
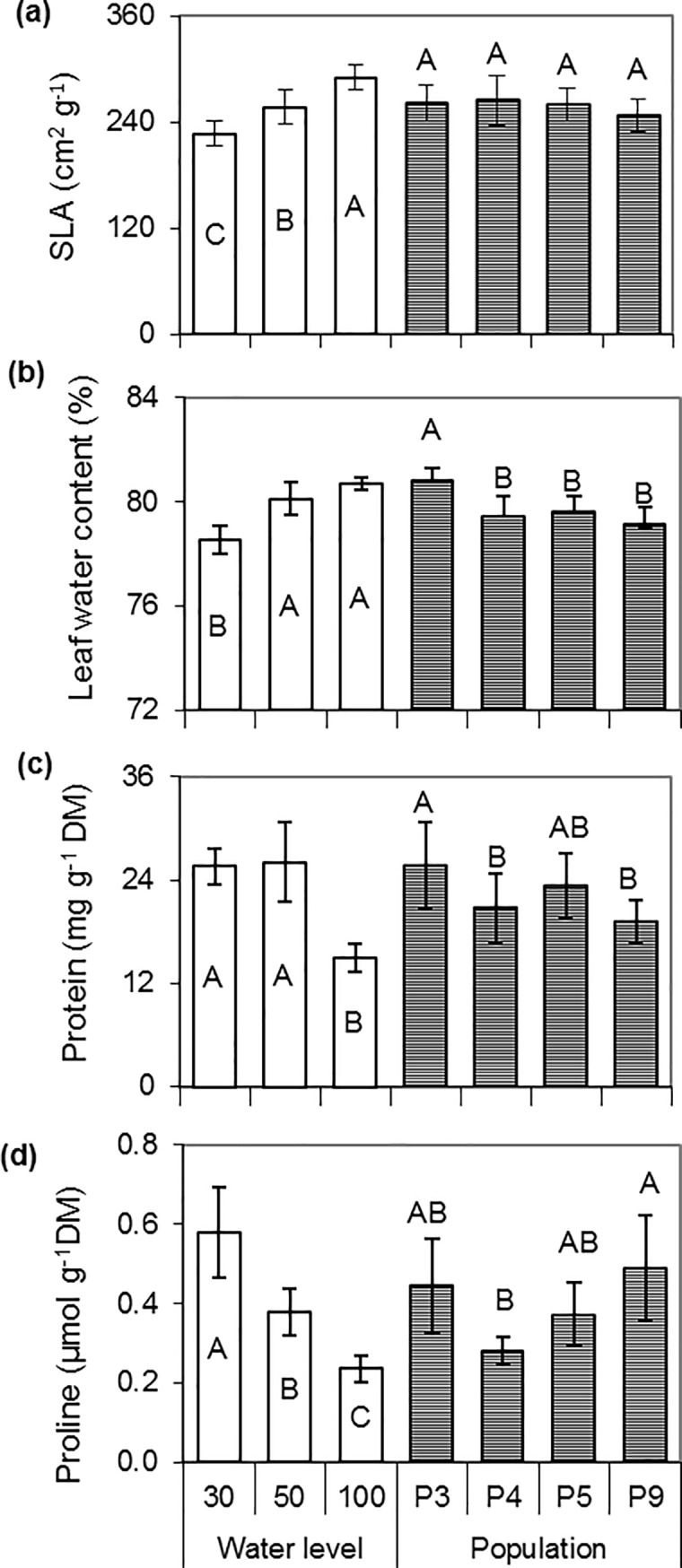
**Variation of specific leaf area (SLA, a), leaf water content (b), soluble proteins (c), and free proline (d) according to water levels and populations.** Bars with different letters show significant differences among means (P < 0.05) for populations (n = 9) or water level (n = 12). The vertical line above the bar indicates the mean standard deviation.

The specific leaf area was positively related to water level, with values that were significantly lower under stronger water stress. There were no differences among populations for this attribute (Fig 5A). Leaf water content (%) was significantly higher in plants under optimal conditions and under moderate stress, while the amount of water retained in the tissues under severe stress was significantly lower. P3 showed significantly more water in the tissues than the other populations (Fig 5B). Moreover, soluble protein concentration was 43% higher in the treatments with water stress (moderate and severe stress), compared to control (100_WL_), with P3 showing the highest protein contents, while P4 and P9 showed the lowest (Fig 5C).

Free proline content significantly increased up to 50% with water stress. The P9 population showed the highest content while P4 showed the lowest proline contents (Fig 5D). The correlation between dry matter and free proline was estimated with a coefficient of -0.58017 (p: 0.0003, N: 36, S2 Fig). Our results also showed a better correlation between dry matter and relative proline content (ProRel), with a coefficient of -0.70797 (p: 0.0001, N: 36, S3 Fig), which is not higher because P4 showed low values of proline at all water levels (S4 Fig).

## Discussion

### Growth and water

In general, the results showed a strong impact of water stress on tall wheatgrass populations. The interaction between water levels and populations revealed differences in dry matter production, with more stable populations, as P5, and less stable ones, as P3.The impact of water level resulted in a decrease in dry matter production, and a decrease in water loss by evapotranspiration with reduced water levels. This was related to increasing water use efficiency under severe stress. The decrease in dry matter was related to reduced leaf size, limiting water loss and photosynthesis, under water deficit [13, 15, 32]. Greater water use efficiency due to increasing stress was also reported for other perennial grasses from dry and/or saline soils [17, 33–34]. To achieve higher water use efficiency under severe water stress, smaller leaf areas and higher photosynthetic activity have been observed [17], maintaining RubisCO and chlorophyll content at healthy levels [33]. However, this advantage could not be maintained if water stress persists, according to Pedrol et al. [27], who found that the strong net photosynthetic rate, of *Holcus lanatus L*. after 45 days of moderate to severe water stress, decreased when the stress persisted for 90 days. Therefore, monitoring photosynthetic rate during longer water stress periods could be of interest for tall wheatgrass under these same water levels.

The leaf water content results showed that *Elymus elongatus* subsp. *ponticus* is a species well adapted to drought conditions, retaining a similar proportion of water in its tissues when grown under optimal conditions or moderate water stress. Leaf water content slightly decreased when plants were grown under severe stress, similarly to what was reported for *Phragmites australis* [33]. As well, relative water content of *Elymus elongatum*, control plants was very stable but it considerably fell due to severe stress [25]. This was also found mentioned for other perennial grasses [17, 35].

### Canopy morphology

The growth of grass in the vegetative can be measured in structural variables of the canopy including leaf size, number and weight of tillers [36]. Increases in water stress determined restrictions to dry matter variables (number and weight of tillers, and length, width and area of leaves). The reduction of leaf size is an early response to water deficit, determined by a lower cell expansion rate [17, 32, 37–39]. In this study, leaf and tiller sizes diminished remarkably with the reduction of water levels, similarly to that reported for other grasses [27, 33–34, 40]. When comparing the values between optimal conditions and severe water stress, the reduction in dry matter and leaf width was lower in this study than for other studies previously reported for another wheatgrass, *Elymus elongatum* [25]. However, the number of tillers was only reduced for some populations under the most severe water stress conditions. This is similar to other perennial grasses, in which reduction of leaf expansion was more pronounced than tillering due to low water potentials [37, 41]. Therefore, the impact of water stress on the reduction of tillering was lower than on biomass production, in concordance with results reported by Durand et al. [38] for *Lolium perenne* and *Festuca arundinacea*. The differential response among the structural variables under water stress suggests that plants give priority to generate individuals at the expense of diminishing their size or/and weight. Therefore, the population with the highest number of tillers would be the most stable in dry matter production.

However, in moderate or no stress conditions, the responses of P3 and P5 populations was very interesting, with similar aerial biomass production levels but different growth strategies, compensating size with tiller density. Similar results of dry matter production with different canopy morphological patterns were also found when comparing tall wheatgrass of different origins in field conditions [20, 22], and between cultivars of other forage species [36].

For these reasons, the interaction between populations and water levels for the production of dry matter cannot be explained with the same canopy morphological patterns. The present study shows that the more stable populations in dry matter production, P5 and P9, are also the populations with lower variations in tiller density in response to water stress, while P3 is the most variable.

To group populations according to the canopy structure would allow selecting materials for different purposes, i.e. the relatively large size of P3 and P4 can make them suitable for the manufacture of hay, while the greater tillering of P5 and P9 can be very useful for grazing pasture.

### Physiological parameters

Lower specific leaf area observed when increasing water stress, as found in our study, was also reported for other grasses [37]. This behaviour could be explained by the reduction of leaf area as a consequence of the inhibition of cell expansion determined by the loss of turgidity and/or elasticity of the cell wall [17, 32, 39]. Up to the first 45 days, smaller cells would be more able to maintain leaf turgidity and photosynthetic activity under severe water stress [27]. Osmotic adjustment is a key mechanism of plants to maintain water uptake and pressure of the cell wall under drought conditions, keeping the stomata open and maintaining photosynthetic rate, leaf expansion, and plant growth [13, 40]. According to that, higher contents of proline and soluble proteins were found in our study when increasing water stress, similarly to reported for other grasses [25, 27, 35, 42].

Water stress usually results in reductions of protein content associated with reduced photosynthetic activity [13, 43]. Therefore, an increase in this parameter suggests tolerance of tall wheatgrass to water stress. The increase of protein content has been associated in *Holcus lanatus* with higher nitrogen reserves, overexpression of RubisCO and the capacity to maintain photosynthetic activity in severe water stress situations [27], while in *Elymus elongatum* it was linked to higher concentrations of heat shock proteins, chaperones and oxidative stress defense enzymes [25].

Proline has been also found to accumulate in many other plant species as a response to stress [12, 16].Some authors have previously mentioned that proline could be in grassesa symptom of damage to the plant [17], while other authors link it to osmoregulation and osmoprotection [25, 35]. The increase in proline content found in our water stress treatments was not due to protein degradation, as protein levels also increased, which is similar to what was reported for other grasses [27, 42]. In this study, the populations with the highest proline concentrations were not always those with the highest dry matter production. In other close wheatgrass, a low relation between saline stress tolerance and free proline content at the intraspecific level was also found [23]. More recent studies suggest that the most important factor for stress protection is the increase in proline biosynthesis rate [16, 35]. This could explain the correlation between relative proline content and dry matter. These results suggest that increased proline accumulation can be an important component of the response of tall wheatgrass to water stress. However, this increase was not strong enough to explain the response to this stress on its own [17, 23], but it could be a variable to be studied to select water stress tolerant germplasm [16] in tall wheatgrass.

### Environment and genetic material

Grass breeding improvement programs seek to increase aerial biomass production and drought tolerance. However, the main mechanism to moderate water use under drought stress is the reduction of plant size, leaf area and tillering, and these mechanisms limit yield potential [15]. Dry matter production of the different populations of tall wheatgrass showed interaction between germplasm and water stress level, which could be related to the adaptation or acclimation of the populations to the original environments. The habitat background of the populations shows different intensity of water stress. Considering the climatic and edaphic environmental conditions such precipitation and irrigation (the latter just in P5), or the availability of water in the soil (higher alkalinity, greater osmotic potential, lower availability), they could be ordered from greater to lower exposure to water stress as follows P4 > P9 > P5 > P3.

P3, collected from humid temperate climates with short summer droughts, showed a great production of dry matter under no or moderate water stress. Its growth strategy was maintaining bigger leaves and tillers size, with high water use efficiency, and the highest levels of proline, protein, and leaf water content. However, P3 had the lowest dry matter and tiller density under severe water stress, suggesting that this population limits, more than other populations, aerial growth to survive under water stress conditions.

P5 was collected at a site close to P4, with semi-arid climate but neutral soil and availability of irrigation water. It showed high aerial biomass production with high water use efficiency at all water levels evaluated. Its growth strategy was maintaining a high tiller density whilst reducing leaf and tiller size, and increasing concentrations of proline and proteins. This suggests that P5 has morphological and physiological mechanisms adapted to grow in situations with and without water stress.

Finally, P4 and P9 were collected from climate-edaphic environments with strong water stress (semi-arid climate in P4 or temperate transitional climate in P9, and both in alkaline soils). These populations could have developed mechanisms to minimize evaporation through different growth strategies (size or density of tillers) and so reduce aerial biomass. This is in contrast to the lowest water use efficiency that we found for these plants. Finally, proline contents were different in P4 and P9, with much higher values in P9 than in P4, suggesting different acclimation mechanisms. This could be due to the fact that P4 was collected in a climate of permanent water stress, while P9 came from an environment with alternating periods of stress. The increase in proline concentration as a strategic mechanism to tolerate stress would be secondary in P4, while in P9, as in the other populations, would be very important.

In summary, the stability in dry matter production in the different populations seems to be connected to the capacity to maintain tillering (high tiller density) under the different situations of water stress. Also, variability in tiller density was characteristic of each germplasm, irrespective of the intensity of water stress of the original environment of the populations.

## Conclusions

*Elymus elongatus* subsp. *ponticus* responded differently depending on the germplasm and the water stress conditions applied during 35 days. The most relevant changes under strong water stress are summarized in a lower aerial biomass production, smaller leaf and tiller sizes, together with a higher water use efficiency, and higher proline and protein contents, with a slight decrease in leaf water content and smaller tiller number in some populations. All these results can be seen as indicators of the tolerance of this species to water stress. However, dry matter and tiller number depended on the population and the level of water stress. At the intraspecific level, the populations differed in their growth strategies and their morpho-physiological mechanisms to survive water stress. This could be related to their habitat backgound, as every population shows different strategies of adaptation or acclimation to new environments. The study shows that greater stability in dry matter production can be related to higher tiller number.

## Supporting information

S1 Table **Dry matter per pot, accumulated evapotranspiration (ET_Accu), water use efficiency (WUE), tiller weight, length, width and area of the leaf (A), leaf water content (LWC), specific leaf area (SLA), proline and protein contents (B), results of two-factor** ANOVA (N = 36). Fixed effects: water levels (WL), populations of tall wheatgrass (Popu) and WLxPopu interaction. Probability values and significance are shown. ET_Accu, proline and protein were transformed (logarithmically) to obtain variance homogeneity and normality.
(PDF)

S2 Table Tiller number per pot, result of two-factor ANOVA with repeated measures (N = 144). Probability values and significance are shown for water levels (WL), populations of tall wheatgrass (Popu), repeated measures over time (t) and their interactions. Data were transformed (logarithmically) to obtain homogeneity of variance.
(PDF)

S1 Fig Tiller number per pot over time (n = 36). **Bars with different letters indicate significant differences among weeks (P < 0.05).** The vertical line above the bar indicate the mean standard deviation.
(TIF)

S2 Fig Proline accumulation as a function of dry matter per pot. Symbols of different colors show different water levels (WL: 100, 50, 30).
(TIF)

S3 Fig Relative proline content as a function of dry matter per pot. Symbols of different colors show different water levels (WL: 100, 50, 30).
(TIF)

S4 Fig Relative proline content as a function of proline accumulation of the control (100WL). Means among populations (P3, P4, P5, P9) under different water levels (WL: 100, 50, 30) are shown (each symbol is the means of n = 3).
(TIF)
