# Supplementary material for: Morpho-physiological responses of tall wheatgrass populations to different levels of water stress
Source: PLoS One. 2018 Dec 17;13(12):e0209281. doi: 10.1371/journal.pone.0209281 (PMC6296543; doi:10.1371/journal.pone.0209281)
Supplement: S1 Table — Dry matter per pot, accumulated evapotranspiration (ET_Accu), water use efficiency (WUE), tiller weight, length, width and area of the leaf (A), leaf water content (LWC), specific leaf area (SLA), proline and protein contents (B), results of two-factor ANOVA (N = 36). Fixed effects: water levels (WL), populations of tall wheatgrass (Popu) and WLxPopu interaction. Probability values and significance are shown. ET_Accu, proline and protein were transformed (logarithmically) to obtain variance homogeneity and normality. (PDF) [file pone.0209281.s001.pdf]

Supporting information

S1 Table. Dry matter per pot, accumulated evapotranspiration (ET_Accu), water use efficiency (WUE), tiller weight, length, width and area of the leaf (A), leaf water content (LWC), specific leaf area (SLA), proline and protein contents (B), results of two-factor ANOVA (N=36). Fixed effects: water levels (WL), populations of tall wheatgrass (Popu) and WLxPopu interaction. Probability values and significance are shown. ET_Accu, proline and protein were transformed (logarithmically) to obtain variance homogeneity and normality.

A) Variable<br>Effect	Dry matter<br>g plot^{-1}	ET_Accu<br>mL H_2O	WUE<br>DMmg H_2OmL^{-1}	Tiller weight<br>g tiller^{-1}	Length leaf<br>cm	Width leaf<br>cm	Area leaf<br>cm^2
WL	<0.0001***	<0.0001***	<0.0001***	<0.0001***	0.0018**	0.0373*	<0.0001***
Popu	0.0029**	0.5845ns	0.0342*	<0.0001***	0.3010ns	0.0358*	0.0098**
WLxPopu	0.0003***	0.0732ns	0.1862ns	0.1359ns	0.8335ns	0.4273ns	0.6273ns

B) Variable<br>Effect	LWC<br>%	SLA<br>cm^2 g^{-1}	Proline<br>µmol DMg^{-1}	Protein<br>mg DMg^{-1}
WL	<0.0001***	0.0001***	<0.0001***	<0.0001***
Popu	0.0002***	0.6232ns	0.0021**	0.0249*
WLxPopu	0.1314ns	0.1521ns	0.2818ns	0.0903ns

ns: non significant P > 0,05, significant at * $P \leq 0.05$; ** $P \leq 0.01$; *** $P \leq 0.001$
