# Supplementary material for: Morpho-physiological responses of tall wheatgrass populations to different levels of water stress
Source: PLoS One. 2018 Dec 17;13(12):e0209281. doi: 10.1371/journal.pone.0209281 (PMC6296543; doi:10.1371/journal.pone.0209281)
Supplement: S2 Table — Probability values and significance are shown for water levels (WL), populations of tall wheatgrass (Popu), repeated measures over time (t) and their interactions. Data were transformed (logarithmically) to obtain homogeneity of variance. (PDF) [file pone.0209281.s002.pdf]

Supporting information

S2 Table. Tiller number per pot, result of two-factor ANOVA with repeated measures (N=144). Probability values and significance are shown for water levels (WL), populations of tall wheatgrass (Popu), repeated measures over time (t) and their interactions. Data were transformed (logarithmically) to obtain homogeneity of variance.

Variable/ Effect	Tillers tillers pot^{-1}
WL	<0.0001 ***
Popu	<0.0001 ***
WCxPopu	<0.0001 ***
t	<0.0001 ***
WLxt	0.4961 ns
Popuxt	0.7221 ns
WLxPopuxt	0.9992 ns

ns: non significant *P* > 0,05, significant at *** *P* ≤ 0.001
