# Supplementary figures and images for: Morpho-physiological responses of tall wheatgrass populations to different levels of water stress
Source: PLoS One. 2018 Dec 17;13(12):e0209281. doi: 10.1371/journal.pone.0209281 (PMC6296543; doi:10.1371/journal.pone.0209281)

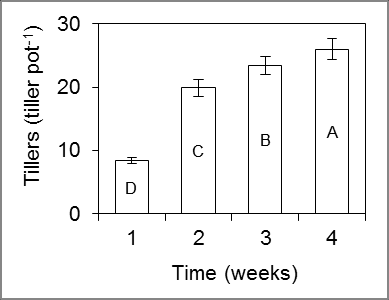

Supplement: S1 Fig — Bars with different letters indicate significant differences among weeks (P < 0.05). The vertical line above the bar indicate the mean standard deviation. (TIF) [file pone.0209281.s003.tif]

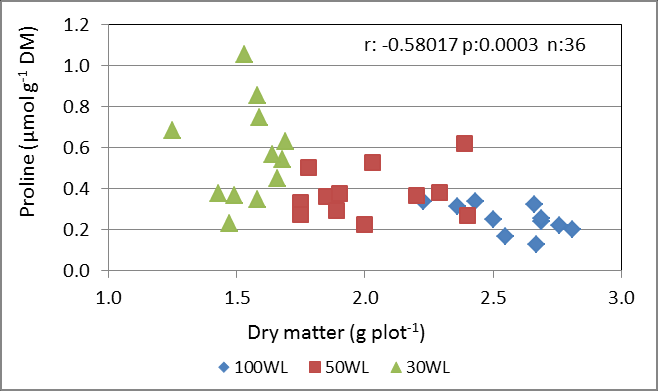

Supplement: S2 Fig — Symbols of different colors show different water levels (WL: 100, 50, 30). (TIF) [file pone.0209281.s004.tif]

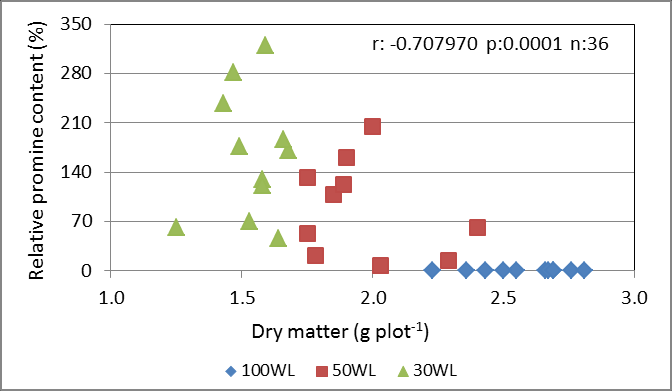

Supplement: S3 Fig — Symbols of different colors show different water levels (WL: 100, 50, 30). (TIF) [file pone.0209281.s005.tif]

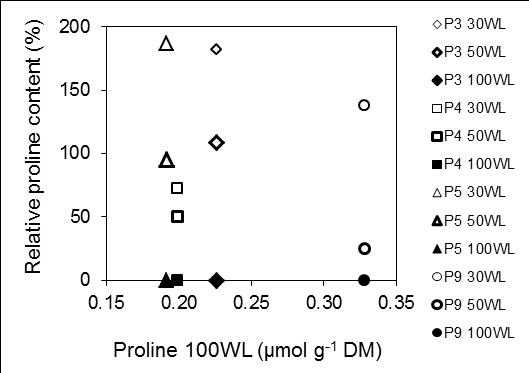

Supplement: S4 Fig — Means among populations (P3, P4, P5, P9) under different water levels (WL: 100, 50, 30) are shown (each symbol is the means of n = 3). (TIF) [file pone.0209281.s006.tif]
